# Complement-Binding Donor-Specific Anti-HLA Antibodies: Biomarker for Immunologic Risk Stratification in Pediatric Kidney Transplantation Recipients

**DOI:** 10.3389/ti.2021.10158

**Published:** 2022-03-16

**Authors:** Vaka K. Sigurjonsdottir, Natasha Purington, Abanti Chaudhuri, Bing M. Zhang, Marcelo Fernandez-Vina, Runolfur Palsson, Neeraja Kambham, Vivek Charu, Kim Piburn, Lynn Maestretti, Anika Shah, Amy Gallo, Waldo Concepcion, Paul C. Grimm

**Affiliations:** ^1^ Division of Nephrology, Department of Pediatrics, Stanford University, Palo Alto, CA, United States; ^2^ Faculty of Medicine, School of Health Sciences, University of Iceland, Reykjavik, Iceland; ^3^ Division of Nephrology, Internal Medicine and Emergency Services, Landspitali–The National University Hospital of Iceland, Reykjavik, Iceland; ^4^ Quantitative Sciences Unit, Department of Medicine, Stanford University, Palo Alto, CA, United States; ^5^ Histocompatibility and Immunogenetics Laboratory, Stanford Blood Center, Stanford University, Palo Alto, CA, United States; ^6^ Department of Pathology, Stanford University, Palo Alto, CA, United States; ^7^ Division of Abdominal Transplantation, Department of Surgery, Stanford University, Palo Alto, CA, United States; ^8^ Transplantation Services, Mohamed Bin Rashid University, Dubai, United Arab Emirates

**Keywords:** antibody-mediated rejection, kidney allograft, children, transplant outcomes, immunosuppression

## Abstract

Antibody-mediated rejection is a common cause of early kidney allograft loss but the specifics of antibody measurement, therapies and endpoints have not been universally defined. In this retrospective study, we assessed the performance of risk stratification using systematic donor-specific antibody (DSA) monitoring. Included in the study were children who underwent kidney transplantation between January 1, 2010 and March 1, 2018 at Stanford, with at least 12-months follow-up. A total of 233 patients were included with a mean follow-up time of 45 (range, 9–108) months. Median age at transplant was 12.3 years, 46.8% were female, and 76% had a deceased donor transplant. Fifty-two (22%) formed C1q-binding *de novo* donor-specific antibodies (C1q-*dn*DSA). After a standardized augmented immunosuppressive protocol was implemented, C1q-*dn*DSA disappeared in 31 (58.5%). Graft failure occurred in 16 patients at a median of 54 (range, 5–83) months, of whom 14 formed *dn*DSA. The 14 patients who lost their graft due to rejection, all had persistent C1q-*dn*DSA. C1q-binding status improved the individual risk assessment, with persistent; C1q binding yielding the strongest independent association of graft failure (hazard ratio, 45.5; 95% confidence interval, 11.7–177.4). C1q-*dn*DSA is more useful than standard *dn*DSA as a noninvasive biomarker for identifying patients at the highest risk of graft failure.

## Graphical Abstract

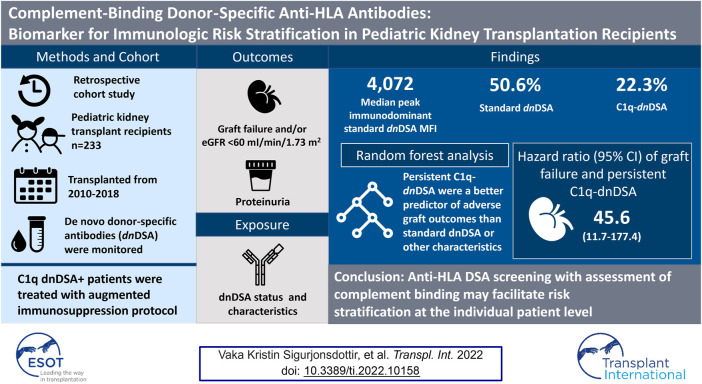

## Introduction

Kidney allograft survival is typically between 10 and 15 years, reflecting an area of unmet need in pediatric kidney transplantation since these children are destined to require more than one transplant during their lifetime. The most common cause of graft failure is induced alloimmune response and rejection (1). Antibodies formed against polymorphic human leukocyte antigen (HLA) molecules on the donor endothelium are central to the pathogenesis of antibody-mediated rejection (ABMR). In kidney allograft recipients, the presence of donor-specific antibodies (DSA) both before and after transplantation correlates with poor graft survival (2–5). DSA that activate the complement system appear to cause particularly severe injury to the allograft, and new complement blood tests (C4d, C1q, C3d) have been developed as tools to stratify the immunologic risk (6–9). Studies link complement-binding *de novo* DSA (C1q*-dn*DSA) to inferior graft outcomes (3, 4, 10–16). The value of using complement-activating antibody testing in clinical care is currently a subject of substantial debate (6, 17–20). In a multicenter study comprising more than 1,000 DSA-positive kidney allograft recipients, Loupy et al. (3) reported that 24% of detected antibodies bound to C1q. More importantly, the glomerular filtration rate (GFR) was lower at 1-year posttransplant, and 5-years graft survival was significantly worse among patients whose DSA-bound C1q as compared to those with DSA that did not demonstrate C1q binding. In a retrospective cohort study of 193 patients at Lucile Packard Children’s Hospital (LPCH) who received a kidney transplant in 2000–2008, a C1q solid-phase assay was employed in parallel to the standard immunoglobulin G (IgG) assay to identify C1q*-dn*DSA (12). Patients with C1q*-dn*DSA (*n* = 15) were almost 6 times more likely to suffer graft failure than those without such antibodies. In fact, 47% of the C1q*-dn*DSA-positive patients suffered premature graft failure at a mean of 33.0 ± 17 months posttransplant. A shortcoming of that study was that C1q*-dn*DSA were analyzed retrospectively in a small cohort using a single blood sample from each case.

A recent study of adult kidney allograft recipients with ABMR showed that persistence of C1q*-dn*DSA after augmented immunosuppressive treatment was an independent determinant of allograft loss (21). We wanted to better characterize the immunologic risk and tailor the treatment to reduce the risk of allograft complications, such as infection, in our pediatric kidney transplant population (22). After reporting that C1q*-dn*DSA are associated with increased risk of premature graft failure in this group of patients (12), we implemented a DSA monitoring and treatment protocol after transplant, using the DSA characteristics and kinetics as a biomarker in an individualized risk stratification for graft failure. Intensified immunosuppressive treatment was guided by the C1q kinetics. The purpose of this study was to assess the performance of our individualized risk stratification using systematic DSA monitoring, including their complement-binding capacity, in addition to the standard approach. We hypothesized that the presence, and in particular persistence, of C1q is a key biomarker for risk stratification.

## Materials and Methods

### Ethical Approval

The present study was approved by the Stanford University Institutional Review Board (49,338). The clinical and research activities reported herein are consistent with the principles of the Declarations of Helsinki and Istanbul.

### Study Population

Pediatric patients who underwent kidney transplantation at Stanford University’s LPCH from January 1st, 2010 to March 1st, 2018 were eligible for the study. Patients with less than 12 months of follow-up, multiorgan transplants, or inconclusive DSA data were excluded. If a patient received more than one transplant during the study period, only data on the first allograft were used.

### Clinical Data

Data were retrospectively extracted from the electronic medical record system, UNOS^®^ and the Stanford Histocompatibility, Immunogenetics, and Disease Profiling Laboratory electronic database at LPCH. Information on patient characteristics, such as cause of end-stage kidney disease, donor information, HLA matching, and age at transplant, immunosuppressive treatment and allograft function were collected. Induction of immunosuppression protocol at LPCH included a rabbit antithymocyte globulin. Maintenance immunosuppression consisted of tacrolimus and mycophenolate mofetil (MMF) for all patients, with or without prednisone, based on immunologic risk. Serum creatinine and urine protein/creatinine ratio were measured at least every 3 months.

### Monitoring, Scoring and Definition of DSA

De novo DSA formation was defined as donor-specific anti-HLA antibodies that were initially identified after the kidney transplant. During the study period, all patients in our kidney transplant program were tested for both standard *dn*DSA and C1q*-dn*DSA at 0 (time of transplant), 1, 2, 3, 6, and 12 months following transplant surgery, at least annually thereafter, and as clinically indicated (e.g., in the case of allograft dysfunction). Information was collected on all *dn*DSA, including A, B, C, DR, DQ and DP specificities. At the Stanford HLA laboratory, commercially available Single Antigen Bead (SAB) assay kits (LAB Screen; One Lambda, Inc., Canoga Park, CA, United States) were used for the detection of antibodies. Standard *dn*DSA were defined as HLA IgG antibodies identified by the solid phase assay on a Luminex platform (23).

The presence of C1q*-dn*DSA was determined using a SAB assay according to the manufacturer’s protocol (C1qScreen™, One Lambda Inc.) on a Luminex platform. In brief, patients’ sera were mixed with polystyrene beads, each uniquely distinguishable by subtle differences in fluorochromes and each coated with a different purified, single‐cloned HLA class I or class II antigen. Data were analyzed using the HLA Fusion™ software (One Lambda Inc.), and interpretations were made using normalized (baseline) mean fluorescence intensity (MFI) values. Cutoffs for positive reactions were >1,000 MFI. DSA were considered to have disappeared if such antibodies were <1,000 MFI at the end of follow-up or at the time of graft failure. Persistence of DSA was defined as any DSA with MFI >1,000 at the end of follow-up or at the time of graft failure, even if they had transiently become negative at any point. Immunodominant DSA (iDSA) was defined as the highest MFI value of standard *dn*DSA. An important methodological issue is how to analyze and report the various patterns of antibody response in a heterogeneous patient group. Some patients may generate an antibody response to 1 or 2 HLA antigens, but with a very high MFI. Other patients might generate antibodies to multiple, even dozens of HLA antigens, but with intermediate or lower MFI. As an approach to this issue we used Jordan’s previously published Relative Intensity Score (RIS) (24–26), in addition to MFI levels. To calculate RIS, we scored combined MFI of all DSA in the following manner: Each DSA with MFI <1,000 received 0 points; MFI 1000–5,000 (weak intensity) received 2 points; MFI 5,000–10,000 (moderate intensity) received 5 points; and MFI >10,000 (strong intensity) received 10 points. The points were summed to form the RIS score.

### Kidney Allograft Biopsies, Definition of Antibody-Mediated Rejection and Immunosuppressive Protocols

During the period of the study, protocol biopsies were performed at 6, 12, and 24 months after kidney transplantation and as clinically indicated (i.e., if graft dysfunction and/or *dn*DSA appearance). To ensure consistency, transplant biopsy specimens were gathered and scored for analysis by an expert in kidney transplant pathology according to the consensus rules of the most recent international Banff Classification criteria (27). Baseline immunosuppression and treatment protocol for ABMR after detection of C1q*-dn*DSA, with or without biopsy, remained unchanged throughout the study period. With the initiation of standardized DSA monitoring, including complement-biding capacity, we implemented an augmented immunosuppressive treatment protocol for all patients with C1q*-dn*DSA MFI >1,000 shown in Table 1. This protocol was unchanged throughout the study period. Patients who formed standard *dn*DSA only were not treated.

**TABLE 1:** Augmented immunosuppressive therapy directed at C1q-*dn*DSA.

• Treatment initiated if complement-binding donor-specific antibodies with MFI ≥1,000 and/or if positive C4d staining on biopsy
• Concurrent cellular rejection treated with corticosteroid if Banff borderline or 1a. Thymoglobulin considered for Banff 1b or 2
• Management
- Tacrolimus target trough levels increased to 7–10 ng/ml and MMF to 4–6 mcg/ml for 2–3 months
- Intravenous immunoglobulin (IVIG) 2 g/kg, administered initially and then every month for minimum of 3 months. Discontinued when C1q-*-dn*DSA MFI <1,000 (defined as disappeared)
- DSA levels are obtained prior to IVIG infusion (data used in study) and immediately after infusion is complete
- If C1q-*dn*DSA persist, can consider continuing IVIG monthly
- If the C1q-*dn*DSA do not respond to IVIG at all after 8 months, discontinuation of treatment should be considered. If MFI levels are decreasing, treatment is continued until disappearance
- Rituximab 500 mg/m^2^ administered within 2 weeks of first dose of IVIG.
- Plasmapheresis added if severe graft dysfunction
- Bortezomib if graft dysfunction is resistant to IVIG and/or plasmapheresis
• After C1q-binding *dn*DSA detection, DSA levels are monitored at a minimum of every 3 months until eliminated. If they do not disappear, DSA levels are followed at least every 3 months for 12 months. C1q-binding *dn*DSA then is monitored based on risk, but at least every 12 months

### Definition of Antibody Status, Risk Factors of C1q-*-dn*DSA Formation and Outcomes

Donor-specific antibody status was considered as the exposure. C1q-*dn*DSA was a time-fixed variable of C1q status, categorized as negative, eliminated or persistent. If C1q-*dn*DSA disappeared after detection at some point and the MFI was <1,000 for the remainder of the study, the C1q-*dn*DSA were considered “eliminated.” In those who continued to have C1q*-dn*DSA throughout the study or at the time of allograft failure, the C1q-*dn*DSA were categorized as “persistent.” Standard *dn*DSA were either negative, present without ever binding complement or detected together with C1q-*dn*DSA. The persistence and elimination of standard *dn*DSA was followed in patients with C1q-*dn*DSA and defined in the same manner as above.

Decreased immunosuppressive therapy as a risk factor for *dn*DSA formation was defined as purposeful reduction of immunosuppression in response to side effects, malignancy or infectious concerns. Nonadherence to medical care was defined when documented in the medical chart, when patients had undetectable tacrolimus levels, missed clinic visits, missed blood draws, or by patient report. Allograft failure was defined as return to dialysis or preemptive re-transplantation. Decreased GFR was defined as estimated GFR <60 ml/min/1.73 m^2^ that persisted over at least 3 months. The creatinine-based “Bedside Schwartz” equation (2009) and/or Chronic Kidney Disease Epidemiology Collaboration equation were used to calculate GFR. Proteinuria cutoff was set at urine protein/creatinine ratio of 0.5 mg/mg that persisted over 3 months. Patients who did not have adverse graft outcome were censored at their last follow up. No patient died before the endpoints of interest were reached.

### Statistical Considerations

Baseline demographic and clinical characteristics of the cohort were summarized descriptively using means (range), medians (interquartile range, IQR), and counts (percentages) as appropriate. Median time-to-allograft failure was reported using Kaplan-Meier estimates. The Kruskal-Wallis, Mann-Whitney, Chi-squared, and Fisher’s exact tests were used to compare clinical characteristics of patients with persistent C1q*-dn*DSA to those of patients with eliminated C1q*-dn*DSA as appropriate based on underlying statistical assumptions. A conditional inference forest analysis was employed to assess the hierarchy of the characteristics of *de novo* anti-HLA DSA based on their ability to predict adverse graft outcome, defined by decreased GFR or allograft failure (28). Included in the model were iDSA MFI, RIS of standard DSA, C1q-binding status, and standard *dn*DSA status. The variables included in the random forest approach were set to zero for patients who did not form anti-HLA *dn*DSA. The conditional forest was fit to 1,000 trees. Given the collinearity among features, conditional variable importance measures were computed in order to quantify the contribution of each variable, using the integrated Brier score as a risk measure (29). Out-of-bag model performance statistics were expressed for the binary outcome of adverse graft event.

Outcomes of graft failure and proteinuria were analyzed based on time-varying C1q*-dn*DSA-binding status. An unadjusted Cox proportional hazards regression model was fit to time-to-allograft failure as a function of time-varying C1q binding. The proportional hazards assumption was assessed for each model and found to hold in all cases (30). Hazard ratios (HR), 95% confidence intervals (CI), and model concordance (using the C-index) are reported (31). Covariates of interest included sex, historic peak panel-reactive antibodies (pPRA), age at transplant, type of donor and HLA match. A *p*-value <0.05 was considered statistically significant. All analyses were conducted using R version 3.5.2.

## Results

### Patient Characteristics

Out of 288 patients who underwent kidney transplantation at LPCH, 233 met the inclusion criteria. One patient who experienced graft failure before 12 months posttransplant due to BK nephropathy was included. Mean follow-up time was 45 (range, 9–108) months. The median age at transplant was 12.3 years, and the majority of the transplant recipients were of Hispanic/Latino origin (39%) and male sex (53%) (Table 2). A little over half (54%) had an HLA match of 0 or 1 out of 6, with only 6% having an HLA match >3/6. The median pPRA was 7%, and 76% of donors were deceased. The most common cause of end-stage kidney disease was renal aplasia/hypoplasia/dysplasia (22%).

**TABLE 2:** Baseline characteristics of pediatric kidney transplant recipients.

	N = 233
Age at transplant, years, median [IQR]	12.3 [11.4]
Sex, *n* (%)	
Female	109 (46.8%)
Male	124 (53.2%)
Race/ethnicity, *n* (%)	
Asian/Pacific Islander	34 (14.6%)
Black or African American	6 (2.6%)
Hispanic/Latino	90 (38.6%)
White	86 (36.9%)
Multiracial	6 (2.6%)
Other	11 (4.7%)
HLA match, *n* (%)	
0–1	125 (53.6%)
2–3	93 (39.9%)
4–6	15 (6.4%)
pPRA, median [IQR]	7.0 [33.0]
Donor status, *n* (%)	
Deceased	176 (75.5%)
Living	57 (24.5%)
Cause of ESKD, *n* (%)	
Renal aplasia/hypoplasia/dysplasia	52 (22.3%)
Glomerulonephritis	33 (14.2%)
Congenital obstructive uropathy	26 (11.2%)
Chronic pyelonephritis (reflux nephropathy)	20 (8.6%)
FSGS	14 (6.0%)
Polycystic kidney disease	11 (4.7%)
Medullary cystic kidney disease	9 (3.9%)
Cortical necrosis	8 (3.4%)
Hemolytic uremic syndrome	5 (2.1%)
Cystinosis	4 (1.7%)
Familial nephritis	4 (1.7%)
Congenital nephrotic syndrome	14 (6.0%)
Other	16 (6.9%)
Unknown	17 (7.3%)

ESKD, end-stage kidney disease; FSGS, focal segmental glomerulosclerosis; HLA, human leukocyte antigen; pPRA, historic peak panel-reactive antibodies; IQR, interquartile range.

### Anti-HLA *dn*DSA Characteristics

**FIGURE 1:**
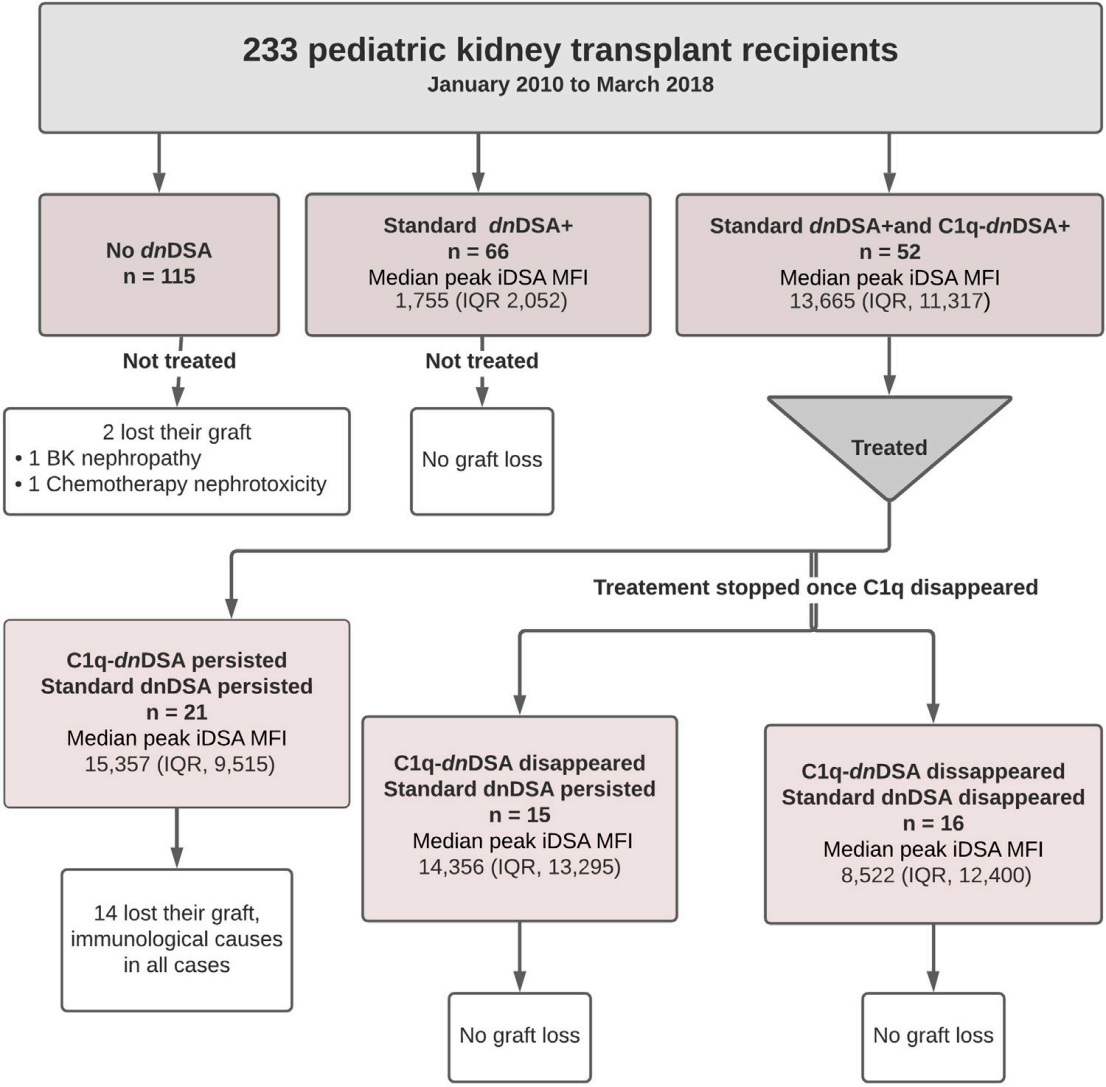
Flow diagram of the study population.

Among the 233 study subjects, 118 formed standard *dn*DSA and of those, 52 also had complement-binding activity triggering interventions at initial detection of C1q-*dn*DSA. A flowchart illustrating the study cohort is provided in Figure 1. C1q-*dn*DSA-directed treatment was individualized based on MFI strength, clinical factors, and biopsy results if available, according to the augmented immunosuppressive therapy protocol. The iDSA were of HLA class 1 in 19 (16.1%) patients and HLA class 2 in 99 (83.8%) patients. The median peak MFI of iDSA in all patients forming anti-HLA *dn*DSA was 4,072 (IQR, 11,304). The iDSA were of DQ or DR specificity in 94 (79.6%) patients. Sixty-six (28.3%) patients formed standard *dn*DSA without C1q binding with a median peak iDSA MFI of 1755 (IQR, 2052), and 52 (22.3%) patients formed C1q*-dn*DSA with the median peak iDSA MFI of 13,665 (IQR, 11,317). The median peak RIS of standard *dn*DSA, calculated for patients who also had C1q-DSA, was 20 (IQR, 22).

**FIGURE 2:**
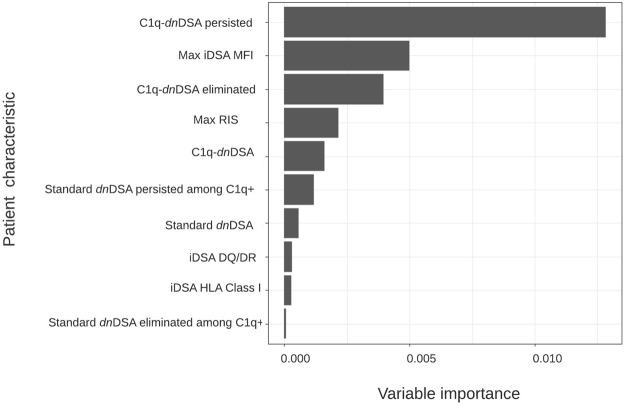
Variable importance plot for time to adverse graft outcome. Hierarchical order of anti–HLA antibody characteristics based on their ability to classify patients according to risk of allograft loss using conditional random forest modeling (*n* = 233).

**FIGURE 3:**
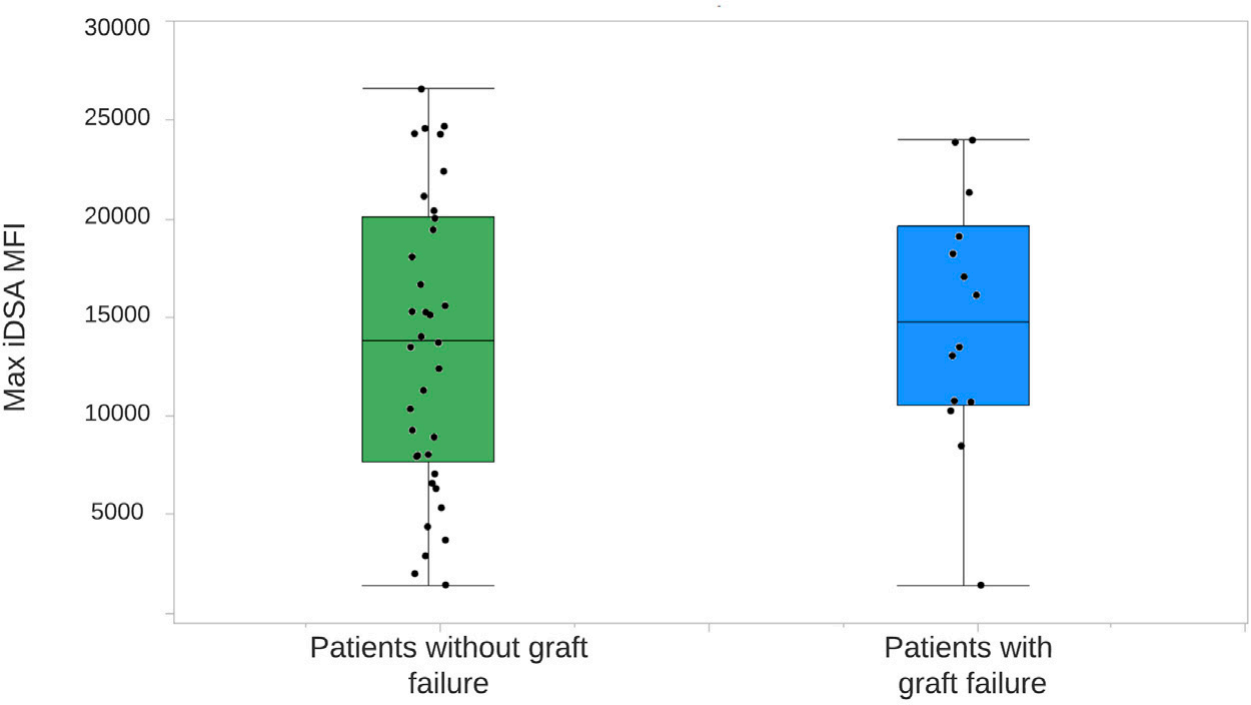
Distribution of iDSA MFI levels in patients forming *dn*DSA, with and without graft loss. The center line represents the median. Each dot represents a single patient. One outlier of max iDSA MFI of 62,344 is not shown among patients without graft failure. iDSA, Immunodominant donor-specific antibody; MFI, mean fluorescent intensity.

All 233 patients were included in the random forest analysis. The most important characteristic for an adverse graft outcome (*n* = 46) was the persistence of C1q binding (Figure 2). Seventeen out of 21 (80.9%) patients with C1q persistence had an adverse graft outcome, 14 of whom lost their graft. In patients without DSA formation, 16 (13.9%) had an adverse graft outcome, including two who lost their graft. Eight (12.1%) of the patients with standard *dn*DSA without complement binding and 5 (16.1%) of those with standard *dn*DSA and disappearance of C1q after treatment experienced an adverse graft outcome, although none lost their graft. Adverse graft outcomes were significantly more frequent among patients with C1q persistence compared to others (*p* <0.0001), but were not significantly different between non-DSA formers, standard *dn*DSA formers only and C1q-*dn*DSA eliminated groups (*p* = 0.86). The random forest model correctly classified only 17 of the 46 (37%) patients as having an adverse graft outcome, while 184 of 187 (98.3%) were correctly classified as not having an event. Of 14 patients who lost their graft due to immunological reasons, 13 had HLA class 2 *dn*DSA. Of those with *dn*DSA and graft failure12 of 14 had DQ- or DR-specific iDSA. Maximum iDSA MFI was more useful to predict graft outcome than maximum RIS. Peak iDSA MFI was correlated with adverse outcome. Looking at the peak iDSA MFI among the 118 patients who formed standard *dn*DSA, patients with adverse graft outcome had significantly higher peak of 10,861 (IQR, 12,394) compared with 2,987 (IQR, 6,891) in those without adverse graft outcomes (*p* = 0.01). If we only analyze the C1q formers, there was no difference between peak iDSA MFI levels in patients with or without graft failure, 13,019 (IQR, 13,665) versus 13,569 (IQR, 7,421), respectively (Figure 3).

### Formation of C1q-*dn*DSA and Antibody-Mediated Rejection

**FIGURE 4:**
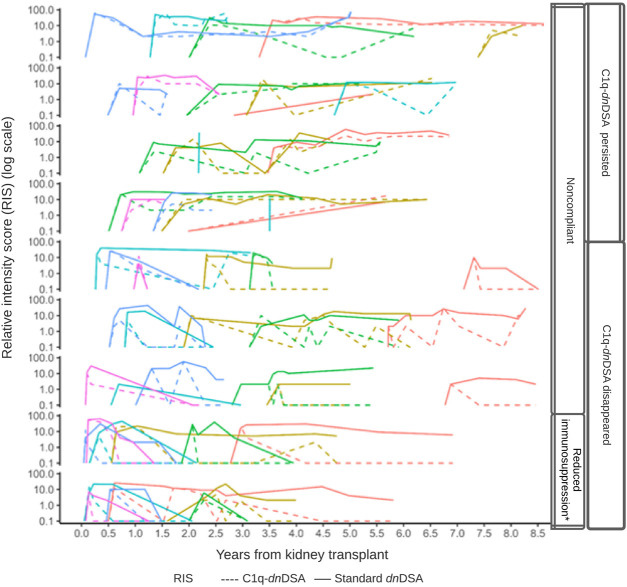
DSA relative intensity score (RIS) over time by C1q status. Line plots of the patients (*n* = 52) with C1q-binding *dn*DSA during the study period. Within each row panel, the colored line corresponds to an individual patient’s RIS trajectory, and the line type corresponds to either C1q-*dn*DSA (dashed) or standard *dn*DSA (solid) RIS. An individual patient’s trajectory does not commence until the first C1q-binding *dn*DSA is detected.

The 52 patients who formed C1q*-dn*DSA had a median time of 3.8 years (IQR, 1.3) until their first detection. C1q*-dn*DSA disappeared in 31 (59.6%) patients and persisted in 21 (40.4%). The time to detection was not different between patients with persistent C1q*-dn*DSA and those who eliminated the antibodies. Forty-four (84.6%) patients had a kidney allograft biopsy carried out at the time of detection, of those 33 (75%) had ABMR according to histological findings. Eight patients were not biopsied, of these four were treated according to protocol without a biopsy, two had contraindications to biopsy, 1 patient refused to have the biopsy performed, and 1 patient started dialysis shortly after detection of C1q. The clinical characteristics and histological findings of patients who formed C1q*-dn*DSA are shown in Table 3. The C1q persistent was more likely to have histological signs of ABMR on biopsy at the time of detection, including positive C4d and interstitial inflammation, compared with the C1q disappearance group. They were also older at kidney transplantation and more likely to be nonadherent to immunosuppressive treatment compared with patients who eliminated C1q. The latter group was younger at transplantation and at first detection of C1q; they were also more likely to have purposeful immunosuppressive therapy reduction as a risk factor for C1q formation. The evolution of DSA over time in the cohort is shown in Figure 4. Standard *dn*DSA RIS and C1q-*dn*DSA RIS were moderately correlated (Pearson correlation coefficient of 0.61). C1q-binding status varied and sometimes reappeared after a period of being undetectable. In the group where C1q disappeared, five patients had at least one C1q reappearance, whereas this was observed for nine patients in the group where C1q persisted. Two patients with C1q-*dn*DSA persistence were treated with plasmapheresis. All patients with persistent C1q had documented medication non-compliance. One patient with persistent C1q had both infections and history of non-compliance at the time of C1q detection. Two patients in whom C1q disappeared did not have an identifiable risk for C1q binding. Comparing the evolution of C1q-*dn*DSA and standard *dn*DSA, the latter stayed elevated longer and did not respond to enhanced immunosuppressive treatment in the same way as C1q-*dn*DSA. Patients with purposeful reduction of immunosuppression appeared to have a steeper decrease of C1q-*dn*DSA. In the patients with C1q-*dn*DSA, persistence of standard *dn*DSA was not strongly correlated with graft failure, unlike C1q persistence. Out of 31 patients who eliminated C1q, 15 (48.4%) had persistent standard-*dn*DSA compared with all 21 patients with persistent C1q*-dn*DSA. Among patients with persistent C1q*-dn*DSA, older age at transplant (HR, 3.69; 95% CI, 1.58–8.63) was significantly associated with graft failure. Younger age at first detection of C1q decreased the risk of graft failure (HR, 0.33; 95% CI, 0.15–0.72). Severe complications after treatment of *dn*DSA, such as malignancies, infections requiring admission to the intensive care unit, or hypogammaglobulinemia requiring intravenous immunoglobulin (IVIG), were not observed.

**TABLE 3:** Clinical and renal histological features of pediatric kidney transplant recipients who formed C1q-*dn*DSA.

Clinical characteristics	C1q disappeared (*n* = 31)	C1q persistent (*n* = 21)	*p*-value
Age at first detection, mean (range)	10 (1–22)	16 (1–24)	0.009
Nonadherence, *n* (%)	18 (58.1)	21 (100)	0.002
Decreased immunosuppressive therapy^a^, *n* (%)	12 (38.7)	1^b^ (4.7)	0.008
Persistence of standard *dn*DSA	15 (48.4)	21 (100)	
Biopsy at the time of first C1q detection, *n* (%)	24 (77.4)	20 (95.2)	
Graft loss, *n* (%)	0 (0)	14 (66.7)	
Age at graft loss, mean (range)	NA	19 (7–24)	
Histological findings (Banff scores)			
Histological diagnosis of ABMR, *n* (%)	14 (58.3)	19 (95)	0.006
C4d	0.6	1.9	0.002
Total inflammation (%)	45.2	66.2	ns
Interstitial inflammation	1.5	2.3	0.04
Tubulitis	1.5	2.1	ns
Interstitial fibrosis	0.9	1.3	ns
Peritubular capillaritis	0.8	1.4	ns
Intimal arteritis	0.2	0.4	ns
Glomerulitis	0.4	0.7	ns
Transplant glomerulopathy	0.21	0.16	ns

a Decreased immunosuppression prescribed by a physician due to side effects, infection or malignancy at the time of C1q detection.

b One patient had both infections and history of medication nonadherence. Scores are presented as means. Banff scores were not significantly different between groups.

### *dn*DSA Characteristics, Graft Failure and Proteinuria

**FIGURE 5:**
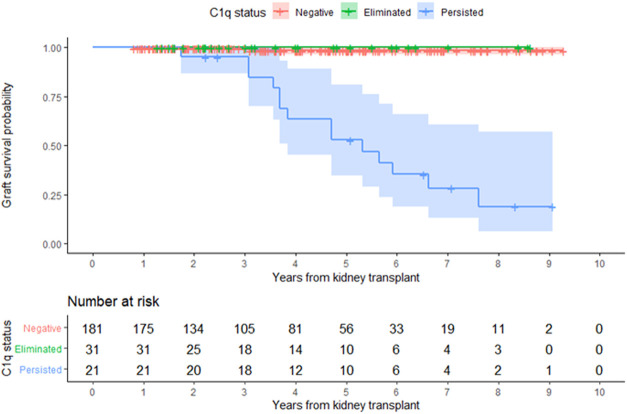
Kidney allograft survival according to overall C1q status. Kaplan-Meier estimate of time to allograft loss.

**FIGURE 6:**
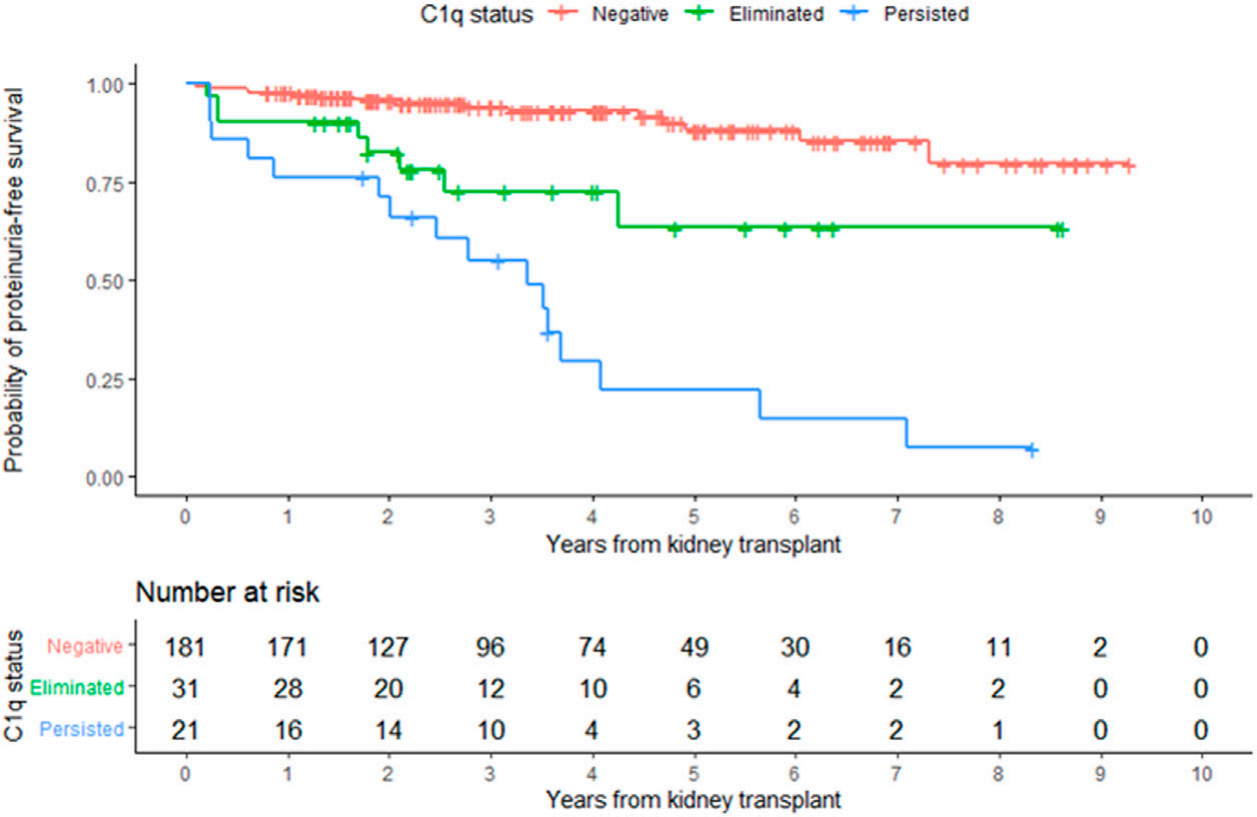
Development of proteinuria according to overall C1q status. Kaplan-Meier estimates of time to proteinuria.

In the entire cohort, graft failure occurred in 16 (6.9%) patients after a median of 45 months (IQR, 28). Fourteen of these patients experienced allograft rejection and two had other potential causes of graft failure; one had BK nephropathy and the other cancer chemotherapy nephrotoxicity (Figure 5). All patients whose grafts failed due to immunological causes had persistent C1q*-dn*DSA in spite of augmented immunosuppressive treatment and lost their graft at a median of 51 months (IQR, 25). Persistent C1q-*dn*DSA were significantly associated with higher risk of graft failure (adjusted HR, 45.46; 95% CI, 11.7 to 177.4). In both the unadjusted and adjusted analysis, elimination of C1q*-dn*DSA was significantly associated with lower risk of graft failure (adjusted HR, 0.02; 95% CI, 0.01–0.09). Among patients with persistent C1q*-dn*DSA, older age at transplant (HR, 3.69; 95% CI, 1.58–8.63) and younger age at first detection of C1q (HR, 0.33; 95% CI, 0.15–0.72) were significantly associated with graft failure. No other demographic or clinical features were associated with graft failure in both groups. There was no correlation between peak iDSA and graft outcome or C1q persistence. The frequency of adverse graft outcome was not significantly different in patients with no *dn*DSA formation, standard-*dn*DSA only and eliminated C1q-*dn*DSA. The risk of proteinuria was highest in patients with persistent C1q*-dn*DSA, intermediate in those with transient C1q, and lowest in those who never developed C1q*-dn*DSA (Figure 6).

## Discussion

In this retrospective study of 233 pediatric kidney transplant recipients, we evaluated the value of systematic anti-HLA DSA screening in predicting risk of adverse graft outcomes and show that compliment binding capacity improved individual risk assessment and is potentially a useful guide of ABMR treatment. Detection of new complement-binding DSA simultaneously with standard *dn*DSA triggered intensified immunosuppression. Patients forming standard *dn*DSA without C1q binding were not treated and had non-inferior outcomes compared with those who did not form *dn*DSA. While higher standard *dn*DSA strength was associated with adverse graft outcomes, no patient experienced graft failure from immunological causes without persistence of C1q-*dn*DSA which was the strongest independent predictor of kidney allograft failure. Furthermore, there was no difference between peak iDSA MFI levels in patients with complement binding DSA with or without graft failure.

The appearance of both new standard *dn*DSA and complement binding serves as a decision point triggering a diagnostic kidney biopsy or a change in therapeutic immunosuppression. The present study shows that C1q-*dn*DSA assessment after interventions may reflect treatment effectiveness and may be a promising approach to guide the intensity and duration of immunosuppressive treatment. C1q-*dn*DSA status after treatment outperformed standard *dn*DSA as a predictor of adverse graft outcomes. Post-treatment assessment of complement binding guides therapy by informing when to stop interventions in case of C1q-*dn*DSA disappearance and justifies continued interventions as long as the C1q-*dn*DSA titer is responding. Additionally, the absence of C1q-*dn*DSA response is a useful sign that should prompt a change or discontinuation of therapeutic interventions. The disappearance of C1q-*dn*DSA is reassuring in our cohort as no patient in that group experienced graft failure. This is in contrast to 66.7% graft failure when C1q-*dn*DSA persists. Our study is in agreement with prior studies demonstrating the association of C1q*-dn*DSA persistence and allograft survival, irrespective of enhanced immunosuppressive therapy (3, 4, 12, 21). Our results, demonstrating that C1q*-dn*DSA response to treatment (persistence or disappearance) is a better predictor of outcome than that of standard *dn*DSA, are similar to those of Ramon et al. in adults treated for severe ABMR with plasmapheresis (16). More severe inflammation and C4d staining at the time of diagnostic biopsy was more common in patients with C1q-*dn*DSA persistence. Our findings expand the results of past research showing that patients with treatment resistant C1q-*dn*DSA were more likely to have a histological diagnosis of ABMR, C4d deposition in peritubular capillaries and interstitial inflammation of the allograft (32, 33). Patients who have resolved their C1q*-dn*DSA might still be at risk for premature graft failure and should be monitored closely. Although this was not apparent in the current study with an average of 45 months of follow-up, graft injury that occurs during the presence of C1q*-dn*DSA may be progressive. The increased frequency of proteinuria in this group supports this notion (Figure 6). We hypothesize that there is a cumulative, dose-dependent effect of nonadherence to immunosuppressive medications on graft survival. Late detection of C1-*dn*DSA or ongoing medication nonadherence after detection may sabotage even the most effective C1q*-dn*DSA elimination therapy.

The strength of anti-HLA antibodies measured with the Luminex technology in the present study correlated moderately with C1q-binding status like most of studies included in a recent meta-analysis (13). The analysis had sufficient power to show that the association of C1q binding and allograft outcomes were independent of the level of standard anti-HLA DSA MFI (13, 32, 34), and that incorporation of complement-binding capacity increased the accuracy of risk prediction above that of the standard anti-HLA DSA MFI levels alone (32). These findings are consistent with the results of the current study. Observing the evolution of MFI of standard *dn*DSA and C1q-*dn*DSA, we noted that standard *dn*DSA MFI tends to stay elevated longer after initiation of therapy. In nearly half of patients with C1q-*dn*DSA disappearance, standard *dn*DSA were not eliminated with none of the patients losing their graft. It has been hypothesized that a difference in the pathogenicity of IgG subclasses may exist in patients with ABMR, explaining variable outcomes in patients with high MFI and rejection (35, 36). IgG is thought to follow a sequential subclass switching after the initial immune response in ABMR (37). The association of persistently elevated titers of standard *dn*DSA with good outcomes in our cohort possibly represent less injurious subclasses of IgG. Peak iDSA or clearance of standard *dn*DSA are not as useful for guiding treatment as C1q-*dn*DSA and would possibly lead to longer and more aggressive treatment regimen, carrying a risk of infection and impacting the cost of therapy.

The study has a several limitations which are largely inherent in the retrospective design. As such, the study was not originally designed for the purpose of addressing biomarker risk prediction or treatment response and therefore weakens the conclusions that can be drawn. Our treatment response has to be interpreted with caution since we did not have a concurrent control group and the two groups might be inherently different. In addition, follow-up allograft biopsy was not obtained in most of the patients. An appropriately powered randomized controlled trial is needed to confirm our observations. One of the strengths of this study is that C1q*-dn*DSA and standard *dn*DSA were simultaneously monitored over a long period of time, allowing us to characterize the kinetics of these antibodies.

In conclusion, this retrospective study of children with a kidney transplant demonstrates that systematic anti-HLA DSA screening together with complement-binding status and allograft biopsies improves risk stratification at the individual patient level and may assist the clinician in determining timing and duration of therapeutic interventions of AMBR. Timely treatment of C1q*-dn*DSA associated ABMR or C1q*-dn*DSA in the absence of kidney allograft biopsy, with early detection and elimination of C1q*-dn*DSA, may be associated with improved outcomes, whereas inability to clear C1q*-dn*DSA identifies the subset of patients with poor graft survival.

## Data Availability

The datasets presented in this article are not readily available because the data cannot be shared due to protection of participant privacy. Requests to access the datasets should be directed to vakaks@gmail.com.

## Abbreviations

C1q-*dn*DSA, C1q-binding *de novo* donor-specific antibodies; *dn*DSA, De novo donor-specific antibody; DSA, Donor-specific antibody; GFR, Glomerular filtration rate; iDSA, Immunodominant donor-specific antibody; IVIG, Intravenous immunoglobulin; LPCH, Lucile Packard Children’s Hospital; MFI, Mean fluorescence intensity; MMF, Mycophenolate mofetil; HLA, Polymorphic human leukocyte antigen; pPRA, Peak panel-reactive antibodies; RIS, Relative intensity score; SAB, Single antigen bead.
